# Extrapulmonary manifestations of severe respiratory syncytial virus infection – a systematic review

**DOI:** 10.1186/cc4984

**Published:** 2006-07-19

**Authors:** Michael Eisenhut

**Affiliations:** 1 Luton & Dunstable Hospital, Lewsey Road, Luton, Bedfordshire, LU4 ODZ, UK

## Abstract

**Introduction:**

Respiratory syncytial virus (RSV) bronchiolitis is the most important cause for admission to the paediatric intensive care unit in infants with lower respiratory tract infection. In recent years the importance of extrapulmonary manifestations of RSV infection has become evident. This systematic review aimed at summarizing the available evidence on manifestations of RSV infection outside the respiratory tract, their causes and the changes in clinical management required.

**Methods:**

Databases searched were Medline (1950 to present), EMBASE (1974 to present), PubMed and reference lists of relevant articles. Summarized were the findings of articles reporting on manifestations of RSV infection outside the respiratory tract in patients of all age groups.

**Results:**

Extrapulmonary manifestations reported in previous observational studies included cardiovascular failure with hypotension and inotrope requirements associated with myocardial damage as evident from elevated cardiac troponin levels (35–54% of ventilated infants), cardiac arrhythmias like supraventricular tachycardias and ventricular tachycardias, central apnoeas (16–21% of admissions), focal and generalized seizures, focal neurological abnormalities, hyponatraemia (33%) associated with increased antidiuretic hormone secretion, and hepatitis (46–49% of ventilated infants). RSV or its genetic material have been isolated from cerebrospinal fluid, myocardium, liver and peripheral blood.

**Conclusion:**

The data summarized indicate a systemic dissemination of RSV during severe disease. Cerebral and myocardial involvement may explain the association of RSV with some cases of sudden infant death. In infants with severe RSV infection cardiac rhythm, blood pressure and serum sodium need to be monitored and supportive treatment including fluid management adjusted accordingly.

## Introduction

Respiratory syncytial virus (RSV) infection is the most common cause of admission to the paediatric intensive care unit (PICU) due to respiratory failure in infancy [1]. Together with influenza virus, RSV is also the most common cause for admissions in adults with chronic cardiac and pulmonary disorders and acute respiratory failure [2]. Extrapulmonary presentations of severe RSV infection were first highlighted in a report on an epidemic affecting infants admitted to a children's hospital in Cleveland (OH, USA). The authors described the features of a 'sepsis syndrome' and noted apnoeas in a significant proportion of infants [3]. Carers need to be aware of manifestations of RSV infection outside the respiratory tract because they may result in otherwise unexpected deteriorations in their patients. For staff looking after the patient with severe RSV infection they may cause both diagnostic and management problems. Awareness of effects of RSV infections outside the respiratory tract are particularly important in managing patients with known underlying comorbidities [2]. It is important to know how much of an organ dysfunction is a temporary effect of RSV or a sign of a deterioration of a pre-existing organ disease, for example in infants with congenital heart disease [4]. This systematic review aims at summarizing evidence on extrapulmonary effects of RSV infection.

## Methods

This systematic review summarizes the findings of articles reporting on manifestations of RSV infection outside the respiratory tract. Included in the analysis were studies in patients of all age groups with RSV infection. Excluded were studies on manifestations that were not specific to RSV but were nonspecific immunological effects of an acute viral infection. A study including data on the influence of respiratory viral infections on nephrotic syndrome [5] was therefore excluded. The databases searched were Medline (1950 to present), EMBASE (1974 to present) and PubMed. Keywords combined for database search were the following: 'respiratory syncytial virus', 'RSV' and 'extrapulmonary', 'paediatric intensive care unit', 'pediatric intensive care unit', 'intensive therapy unit', 'intensive care unit', 'myocardium', 'myocardial', 'arrhythmia', 'inotropes', 'shock', 'cardiac failure', 'hepatitis', 'apnoea', 'seizure', 'fit', 'hyponatremia', 'hyponatraemia' 'antidiuretic hormone', 'kidney', 'CSF', 'cerebrospinal fluid'. Reference lists of relevant articles were also searched.

## Results

### RSV and the cardiovascular system

The first report of clinically symptomatic myocardial involvement during RSV bronchiolitis was that of a case of fatal interstitial myocarditis in a child in 1972 [6]. Other early reports include also the development of a second-degree heart block during the disease. Subsequently a report of an RSV-associated multifocal atrial tachycardia appeared, a phenomenon that was again reported in a later series of patients with RSV-associated atrial tachycardias [7,8]. Other forms of supraventricular tachycardias have also been reported during RSV infection; they seemed to occur in patients with structurally normal hearts and were not associated with hypoxia or beta-agonist therapy [8,9]. Life-threatening arrhythmias have also been reported. Atrial flutter was associated with cardiogenic shock in one patient. This previously healthy patient had also had long runs of ventricular tachycardia including torsades de pointes. Ventricular fibrillation developed after an attempt at overdrive pacing [10]. Another case of ventricular tachycardia requiring cardioversion was reported subsequently [11]. Another life-threatening complication can be cardiac tamponade evolving from pericardial effusion [10,12].

Cardiovascular compromise in the form of hypotension without cardiac arrhythmias has also been described and has been associated with evidence of myocardial damage as indicated by elevated cardiac troponin I and T levels. Features of shock have first been described in seven infants in an observational study on 218 infants admitted to a children's hospital [3]. Elevated cardiac troponin levels were found in 35 to 54% of infants with RSV infection ventilated in PICUs [13,14]. Elevated cardiac troponin I levels have also been found in children with RSV infection not requiring mechanical ventilation [15]. The degree of cardiovascular support described ranged from the administration of fluid boluses [14] to inotropic support [13,16].

The first report on detection of RSV in the myocardium in patients with bronchiolitis was in an infant with combined immunodeficiency; the virus was cultured from myocardium [17]. More recently RSV was again detected in the myocardium by PCR in a patient with myocarditis [18].

### Central nervous system manifestations of RSV

Acute neurological signs and symptoms such as central apnoeas, seizures, lethargy, feeding or swallowing difficulties, abnormalities of tone or strabism, abnormalities of the cerebrospinal fluid (CSF) or the electroencephalogram were found in 39% (*n *= 121) of RSV-positive patients on a PICU [19]. In a population of RSV-positive patients admitted to the general paediatric ward – that is, with milder disease and excluding patients with simple febrile convulsions – neurological complications were found in 1.2% of patients (*n *= 964) [20]. Looking at the occurrence of seizures as a manifestation of an encephalopathy, another group found an incidence of seizures of 1.8% (*n *= 487) in patients with RSV bronchiolitis admitted to a paediatric tertiary referral centre [21]. RSV was detected by RT-PCR in the CSF of a four-month-old infant with apathy and what seemed to be a febrile convulsion [22]. CSF abnormalities were found in one study in 12 of 30 RSV patients who had a lumbar puncture [19]. Other studies have found antibodies specific for RSV in the CSF of patients with RSV bronchiolitis [23,24].

#### Central apnoeas

Apnoeas defined as a respiratory arrest for more than 20 seconds and/or bradycardia with accompanying cyanosis or oxygen desaturation below 90% have been found in 16 to 21% of children admitted to hospital with RSV infection [25]. The most important risk factor associated with apnoeas was age below two months. Apnoeas on admission increased the risk for recurrent apnoeas, and these children did have a significantly increased probability of requiring mechanical ventilation [25]. A prospective experimental study in infants investigated apnoea responses in infants with RSV infection. RSV-infected infants were compared with non-infected infants with regard to the reflex apnoea response to sterile water instilled into the pharynx (laryngeal chemoreflex) during sleep. The data were put into relationship to nasopharyngeal levels of IL-1β and IL-6. Both the duration of the first apnoea and the total apnoea duration (all apnoeas) were significantly longer in patients with RSV than in controls (see Table 1). There was a significant negative correlation between nasopharyngeal IL-1β levels and the duration of apnoea [26]. The apnoeas were not associated with a higher level of pro-inflammatory cytokines.

#### Seizures

Seizure types found to be associated with RSV infection include both generalized tonic–clonic and partial seizures with altered consciousness and focal motor features or eye deviation [19,20]. They were found in 0.7% (admissions to the ward) to 6.6% (admissions to PICU) of patients. Some patients presented with a status epilepticus [20]. Abnormalities in the electroencephalogram have been noted in some patients [19]. A cause of seizures in infants with RSV infection previously identified is hyponatraemia [27] (see below under endocrinological manifestations of RSV infection).

#### Other neurological manifestations

Strabismus has been reported as a neurological complication in two large studies [19,20]. It was found in the form of esotropia in four of 12 patients with neurological complications [20]. One case of acute axonal polyneuropathy [19] and a case with features of encephalitis on imaging with magnetic resonance imaging and positron emission tomography scanning have also been described [28]. Diaphragmatic flutter characterized by involuntary high-frequency contractions of the diaphragm, occurring at a rate of 150 to 480 contractions per minute asynchronous with the heartbeat, has been discovered by chance on review of recordings from respiratory inductive plethysmographs and impedance pneumographs in three infants with RSV infection who were extensively monitored because of concerns about apnoeas [29]. Diaphragmatic flutter has been associated with inability to wean patients from mechanical ventilation as well as with the need for assisted ventilation in adults [30].

### Endocrine effects of RSV bronchiolitis

#### Antidiuretic hormone

Hyponatraemia (a serum sodium level of less than 136 mmol/l) was found in 33% of infants requiring intensive care with RSV infection; 11% had a serum sodium level of less than 130 mmol/l [27]. In a less selected population of children, including patients with milder disease, only 0.6% of patients had a serum sodium level of less than 130 mmol/l [31]. This phenomenon has prompted investigations into the underlying endocrine causes. The first report on investigations of hyponatraemia associated with RSV infection dealt with four infants admitted to the ward with hyponatraemia and bronchiolitis during an outbreak of RSV. One presented with focal seizures and hyponatraemia and was found to be RSV positive. All four infants had elevated antidiuretic hormone (ADH) levels. One had a synacthen test done, which showed normal cortisol release [32]. Further investigations revealed that ADH levels were significantly higher in patients with bronchiolitis than in patients with apnoeas or upper respiratory tract infections with RSV. The highest levels were found in patients receiving mechanical ventilation [33]. Increased ADH levels were associated with high arterial partial pressure of CO_2 _and hyperinflation on a chest X-ray. There was, however, no association between ADH levels and serum sodium levels in this study. Hyponatraemia and hyponatraemic seizures have in this context been associated with the application of hypotonic fluids at 100 to 150 ml/kg per day [27].

#### Stress hormone responses

A prospective study comparing ventilated infants with RSV infection and patients admitted to the ward showed that ventilated patients had higher prolactin and growth hormone levels and significantly lower leptin and insulin-like growth factor-1 levels. Cortisol levels were not different. The leptin and prolactin levels accounted for 57% of the variation in lymphocyte count, which was significantly lower in ventilated patients with RSV infection [34].

### Respiratory syncytial virus-associated hepatitis

**Figure 1:**
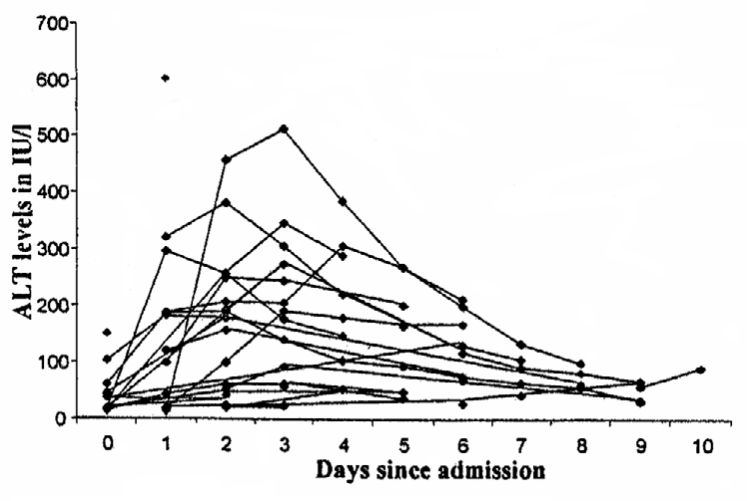
Alanine aminotransferase (ALT) levels in infants with respiratory syncytial virus infection ventilated on a paediatric intensive care unit.

Elevated transaminase levels have been found in 46 to 49% of ventilated children with RSV bronchiolitis [35,36]. Severe hepatitis with alanine aminotransferase levels of nearly 3,000 IU/l has been noted and this was associated with coagulopathy [35]. The peak of transaminase levels was found to be between two and four days after admission (see Figure 1). Respiratory disease, as judged by duration of ventilation, was more severe in infants with elevated transaminase levels [35,36]. The prevalence of hepatitis was 80% in children with congenital heart disease. This was a significantly higher prevalence than the one found in children without congenital heart disease [36]. Direct invasion of the liver in an immunocompetent infant with RSV infection has been documented by the successful culture of RSV from material of a liver biopsy [37]. Hepatic involvement in the form of fatty changes was described in a fatal case of Reye's syndrome associated with RSV infection [38].

### Other extrapulmonary manifestations of RSV bronchiolitis

Several other extrapulmonary manifestations of RSV infection have been described but most of them only in single case reports and none of them seem to be life threatening. They include hypothermia [3], exanthems involving the trunk and face in the form of a finely granular, scarlatiniform rash [3,39], thrombocytopenia and conjunctivitis [40].

### Supportive management of extrapulmonary manifestations of respiratory syncytial virus infection

Previous case series showed that RSV-associated ventricular arrhythmias may respond to antiarrhythmic drugs such as lidocaine and beta-blockers, and cardioversion [10]. Hypotension may respond to simple fluid resuscitation [14] and, if this is not successful, inotropic support for a few days [16].

Strategies used to treat RSV-associated apnoeas in previous studies, none of which were randomized controlled trials, included loading with caffeine, nasal continuous positive airway pressure, negative-pressure ventilation, and intubation and mechanical ventilation [41,42]. Loading with caffeine significantly reduced the frequency of apnoeas in seven infants with RSV infection. Hyponatraemic seizures have been managed successfully and safely by increasing the sodium levels of less than 25 mmol/l over 48 hours (about 0.5 mmol/l per hour). Hyponatraemic seizures may be resistant to anticonvulsive therapy and may require a more rapid correction by a 3% saline bolus of 3 to 5 ml/kg followed by fluid restriction [27]. Hepatic involvement should prompt the clinician to investigate for structural heart disease causing ischaemic hepatitis.

## Discussion

Extrapulmonary manifestations suggest that RSV may infect organs other than the lung. It is unlikely that systemic co-infection with bacterial pathogens is responsible for most extrapulmonary manifestations. Previous studies have shown that serious bacterial infection is present in 0.6 to 1.2% of children admitted with RSV infection [43]. A previous study found that in 63% of neonates and in 20% of infants with RSV detected in nasopharyngeal aspirate on the PICU, RSV RNA was detectable in peripheral blood by nested RT-PCR [44]. The detection of RSV RNA in arterial blood (four infants with bronchiolitis) was also reported by another group [45]. These findings demonstrate the way in which RSV is carried to extrapulmonary sites. It can be postulated that apnoeas and arrhythmias have led to unexpected deaths in infants with RSV disease in the community, even though the detection of RSV nucleic acid in postmortem tissue of infants who died of sudden infant death syndrome was not more common than in infants who died from unrelated causes during the same time period [46].

### RSV and the cardiovascular system

Some of the authors of reports on arrhythmias or myocardial failure in RSV infection doubted a direct role of the virus. As highlighted in a previous report [47], right ventricular decompensation due to pulmonary hypertension is a possible cause for myocardial damage, cardiac troponin elevation and systolic hypotension. Pulmonary disease is associated with pulmonary hypertension in bronchiolitis [48]. Cardiac troponin T elevation has previously been reported in patients with bacterial pneumonia [49]. Right ventricular strain may also precipitate arrhythmias [50]. However, a direct involvement of RSV is suggested by its isolation from myocardial tissue and the reported occurrence of significant pericardial effusion.

### RSV and the central nervous system

Apnoeas were the most common neurological manifestation of RSV infection. A prospective experimental study looking at the laryngeal chemoreflex [26] has clearly demonstrated that there is an abnormal response at the level of the central nervous system involved rather than that the apnoeas are secondary to respiratory compromise or seizures alone. Detection of RSV in the CSF has also supported a direct invasion of the central nervous system in RSV disease.

### RSV and the endocrine system

The lack of association of ADH levels with the reduced serum sodium levels may be due to the associated hyperreninaemia and features of secondary hyperaldoseronism leading to sodium retention found in another study [51]. One can speculate that perceived hypovolaemia by intrathoracic receptors may be involved. It seems that the development of hyponatraemia requires the presence of both raised ADH levels and a source of electrolyte-free water [27]. The study looking at the neuroendocrine stress response found that, in keeping with previous studies, lymphopenia is not related to increased cortisol levels and provided new data on a possible relationship of the low lymphocyte counts with increased prolactin and low leptin levels. There is good evidence for a role of leptin in the prevention of stress-induced apoptosis of T lymphocytes [52].

### RSV and the liver

The higher prevalence of hepatitis in children with congenital heart disease may indicate that hepatic venous congestion as a result of right ventricular failure causing ischaemic hepatitis may be involved in transaminase elevation in some of these children. Apart from the documented direct invasion of the liver, a possible effect of cytotoxic CD8-positive T lymphocytes not requiring the presence of RSV itself has recently been implicated in collateral damage to the liver in mild influenza virus infection [53].

### Agenda for future research

Future research needs to include randomized controlled trials on the treatment of RSV-related central apnoeas by medication such as caffeine, which may be able to prevent the need for mechanical ventilation. There is a lack of data on extrapulmonary manifestations of RSV infection in the elderly where co-morbidities such as ischaemic heart disease and cerebrovascular insufficiency may put them at higher risk of their complications. Future studies need to clarify how common extrapulmonary manifestations such as arrhythmia and hepatitis are in patients with mild RSV infection. Long-term follow-up case control studies, including detailed neuroimaging, of infants with acute neurological manifestations of RSV infection need to clarify whether there are potential long-term sequelae.

## Conclusion

Extrapulmonary manifestations are common in ventilated infants with severe RSV infection. This systematic review highlights that cardiac rhythm and blood pressure need to be monitored carefully in these patients, to detect potentially life-threatening complications (Table 2). Plasma sodium levels need to be checked daily in patients requiring intravenous fluids, and fluid input needs to be adjusted to avoid the development of hyponatraemia and the associated seizures. These requirements should be balanced against potential complications of invasive monitoring and overtreatment of infants with RSV infection on the PICU, which have been found to be associated with increases in costs and morbidity without improvement in outcome [54].

## Key messages

• 	Extrapulmonary manifestations are common in children with severe RSV infection.

• 	Life-threatening extrapulmonary manifestations of RSV infection include central apnoeas, status epilepticus, ventricular tachycardias and fibrillation, heart block and pericardial tamponade and can be detected by adequate monitoring.

• 	RSV-associated hyponatraemia is common, can cause seizures and needs to be treated by adequate fluid management.

## Abbreviations

ADH = antidiuretic hormone; CSF = cerebrospinal fluid; PICU = paediatric intensive care unit; RSV = respiratory syncytial virus; RT-PCR = reverse transcriptase polymerase chain reaction.

## Competing interests

The authors declare that they have no competing interest.

## Authors' contributions

The author is the designer and sole contributor to this work.

## Figures and Tables

**Table 1:** Duration of apnoeas in response to the laryngeal chemoreflex in infants

Status	Duration (s)		
	First apnoea	Total apnoea	Recovery time
RSV positive	5.0 ± 0.7^a^	10.9 ± 1.8^a,b^	38.0 ± 6.0^a^
RSV negative	3.1 ± 0.5	5.3 ± 1.0	21.0 ± 0.9
Controls	2.5 ± 0.4	3.4 ± 1.1	19.0 ± 5.0

The table is taken from [25], with the permission of Taylor & Francis.

Results are given as means ± SEM. RSV, respiratory syncytial virus; RSV negative, patients with RSV-negative bronchiolitis; controls, patients without respiratory tract infection. ^a^*p *< 0.05 between RSV-positive patients and controls; ^b^*p *< 0.05 between RSV-positive patients and RSV-negative patients.

**Table 2:** Life-threatening extrapulmonary complications of severe infection with respiratory syncytial virus

Organs affected	Complication	References
Brain	Apnoeas; status epilepticus	[19,20]
Heart	Ventricular tachycardia; ventricular fibrillation; cardiogenic shock; complete heart block; pericardial tamponade	[7,10,12]
Brain, liver and kidney	Reye's syndrome	[38]
